# cMYC expression in thyroid follicular cell-derived carcinomas: a role in thyroid tumorigenesis

**DOI:** 10.1186/s13000-017-0661-0

**Published:** 2017-10-03

**Authors:** Hany I. Sakr, Deborah J. Chute, Christian Nasr, Charles D. Sturgis

**Affiliations:** 1 Cleveland Clinic, Department of Pathology and Laboratory Medicine, 9500 Euclid Avenue, L25, Cleveland, OH 44195 USA; 2 Cleveland Clinic, Department of Endocrinology, Diabetes and Metabolism, Cleveland, USA

**Keywords:** Thyroid carcinoma, cMYC, Immunohistochemistry, Tumorigenesis, Tissue microarray, Formalin-fixed, Paraffin-embedded

## Abstract

**Background:**

*cMYC* regulates approximately 15% of human genes and is involved in up to 20% of all human cancers. Reports discussing cMYC protein expression in thyroid carcinomas are limited, with controversies pertaining to cMYC expression patterns noted in the literature. The aims of the current study were to clarify patterns and intensities of cMYC expression in follicular cell-derived thyroid carcinomas across a spectrum of cancer morphologies and disease aggressivities, to correlate cMYC with BRAF^V600E^ expression, and to evaluate the potential role of cMYC in progression of well-differentiated thyroid carcinomas into less well-differentiated carcinomas.

**Methods:**

Immunohistochemical studies using specific monoclonal antibodies for cMYC and BRAF^V600E^ were performed on tissue microarrays built from follicular cell-derived thyroid carcinomas (25 papillary, 24 follicular, 24 oncocytic variant of follicular, and 21 undifferentiated). In addition, cMYC IHC testing was also performed on whole tissue tumor sections from a subset of patients. Nodular hyperplasia cases were used as non-neoplastic controls. Appropriate positive and negative controls were included.

**Results:**

cMYC was expressed almost exclusively in a nuclear fashion in both thyroid carcinomas and nodular hyperplasias. cMYC expression was weakly positive in both nodular hyperplasias and well-differentiated carcinomas. The majority of undifferentiated carcinomas (UDCs) showed strong nuclear cMYC positivity. PTC cases that were positive for cMYC (6/25) harbored the *BRAF* ^*V600E*^ mutation. A correlation was confirmed between cMYC intensity and tumor size in UDCs. UDC cases that developed out of well-differentiated thyroid carcinomas showed frank overexpression of cMYC in the undifferentiated tumor components.

**Conclusions:**

Our study suggests that nuclear overexpression of cMYC correlates with tumorigenesis / dedifferentiation in follicular cell derived thyroid carcinomas, a concept that has not been shown before on whole tissue sections.

## Background

Thyroid cancer is the most common malignancy of the endocrine system. Approximately, 1.1% of men and women will be diagnosed with thyroid cancer at some point during their lifetimes [1]. Carcinomas derived from thyroid follicular epithelium comprise papillary, follicular, poorly differentiated, and undifferentiated (anaplastic) carcinomas. Understanding the molecular genetic alterations that drive thyroid carcinogenesis is important and may have prognostic and therapeutic implications, especially in poorly differentiated tumors. Some studies have linked certain oncogenes as well as tumor suppressor genes to the progression of well-differentiated thyroid carcinomas (papillary and follicular) into less differentiated ones (poorly differentiated and anaplastic) [2–6]. However, other studies have failed to show such correlations, questioning the tumor progression model that is well characterized in other organs systems [7, 8].

*cMYC* is a proto-oncogene located at chromosome 8q24.1. It encodes a nuclear phosphoprotein that acts as a growth promoter and a transcription factor. *cMYC* regulates approximately 15% of human genes and is estimated to be involved in 20% of all human cancers [9]. The available literature discussing cMYC expression in thyroid follicular cell-derived carcinomas focuses mainly on gene and/or mRNA expression levels [1–13]. Reports discussing cMYC protein expression in thyroid carcinomas are limited, and controversies exist pertaining to cMYC expression patterns (nuclear versus cytoplasmic immunoreactivity) in these tumors. Most authors report nuclear overexpression of cMYC to correlate with tumourigenesis; however, a few early studies also reported cytoplasmic expression (likely secondary to subcellular localization) [14–20]. Table 1 summarizes relevant IHC studies detailing cMYC expression in thyroid follicular cell-derived carcinomas [14–18, 21–24]. Herein, we utilized IHC for cMYC in a tissue microarray (TMA) study performed on a spectrum of follicular cell-derived thyroid carcinomas, including papillary (*n* = 25), follicular (*n* = 25), oncocytic variant of follicular (*n* = 25), and undifferentiated (*n* = 22) carcinomas. Twenty five cases of thyroid nodular hyperplasia were also included in the TMA as non-neoplastic controls. Aims of this study were to further clarify patterns of cMYC expression in thyroid carcinomas across the spectrum of morphology and disease aggressivity, to evaluate potential cMYC diagnostic usefulness, to find a correlation (if any) between cMYC and BRAF^V600E^ expression in thyroid carcinomas, and to determine the extent to which cMYC may contribute to carcinogenesis.

**Table 1:** Summary of **cMYC** expression studies (using IHC) in thyroid follicular cell-derived carcinomas

Tissue Studied	PTC (% Sn, n)	FC (% Sn, n)	OvFC (% Sn, n)	UDC (% Sn, n)	Staining pattern	*Ref.*
Polyclonal antibody						
TB	100 (27)	100 (5)	ND	ND	C	Mizukami et al.,^20^ 1991^a^
TMA	?? (44)	?? (35)	ND	?? (11)	C, N^b^	Braunschweig et al.,^21^ 2007
Monoclonal antibody						
TB	90 (39)	67 (16)	ND	100 (6)	C	Hashimoto et al.,^22^ 1990
TB	ND	45 (20)		ND	C	Auguste et al.,^23^ 1992^c^
TB	ND	17 (12)	100 (8)	ND	C	Masood et al.,^24^ 1993
TB	100 (19)	100 (4)	ND	100 (2)	N	Haugen et al.,^27^ 1993^d^
TB	ND	ND	ND	59 (22)	N	Kurihara et al.,^28^ 2004
TMA	66 (167)	ND	ND	ND	N	Lee et al.,^29^ 2012^e^
TB	81 (58)	ND	ND	ND	N	Hu et al.,^30^ 2015

The biomarkers are arranged according to the type of antibody used; polyclonal followed by monoclonal. For each histologic type of thyroid follicular cell-derived carcinoma, the sensitivity of the biomarker studied is documented first (indicated by % of immunoreactivity reported in the study) followed by the total number of cases studied. Any percentage with decimal ≥0.5 is rounded up to 1, otherwise rounded down

^a^cMYC immunoreactivity was variable in PTC and FC. The antibody used showed immunoreactivity in all follicular adenomas and Grave’s disease cases tested as well

^b^Antibody’s immunoreactivity signals were quantified digitally using P-SCAN software

^c^The 20 cases included FC and OvFC without clear distinction

^d^cMYC immunoreactivity was weak in FC and 50% of PTC cases. The antibody also showed immunoreactivity in all follicular adenomas and Grave’s disease cases

^e^cMYC immunoreactivity was variable

*IHC* immunohistochemistry, *NH* nodular hyperplasia, *PTC* papillary thyroid carcinoma, *FC* follicular carcinoma, *OvFC* oncocytic variant of follicular carcinoma, *UDC* undifferentiated carcinoma, *TB* tissue blocks, *TMA* tissue microarray, *ND* not done, *Sn* sensitivity, *C* cytoplasmic, *N* nuclear

## Methods

### Patient selection

The Cleveland Clinic anatomic pathology archives were reviewed to identify patients diagnosed with thyroid follicular cell-derived carcinomas. A spectrum of carcinoma cases was selected from archival material. The study set included 25 cases of papillary thyroid carcinoma (PTC), as appearing consecutively in the retrieval, and including cases with follicular, classical, oncocytic and tall cell features (2011 archive). In addition, 25 consecutive cases of follicular thyroid carcinoma (FC) (2004-2014 archive), and 25 consecutive cases of the oncocytic variant of follicular carcinoma (OvFC) (2005-2014 archive) were retrieved. The archive was searched for the period from 2004 to 2014 and only 22 consecutive cases of undifferentiated carcinoma (UDC) were found. In addition, 25 cases of nodular hyperplasia (NH) were included as controls (2011 archive). We did not include poorly differentiated carcinomas (PDC) in our TMA study as our data retrieval did not provide enough pure cases to allow for pair-wise correlations with the number of cases in the other diagnostic categories. The majority of poorly differentiated carcinomas in our archive existed as focal areas of disease in backgrounds of better differentiated carcinomas, and did not lend themselves as readily to study by TMA as undifferentiated tumors with large (whole tissue block) areas of anaplastic malignancy.

Clinical details were obtained from patients’ pathology reports and original electronic medical records. The morphologic features, histopathologic variants, and the extent of tumor at time of diagnosis (pT) were classified according to World Health Organization (WHO) criteria and American Joint Committee on Cancer (AJCC) parameters [25, 26]. Thirteen UDC cases (59% of 22) were documented to be associated with either concomitant (11/13) or pre-existing (2/13) well differentiated thyroid carcinomas [11/13 (85%) PTC, and 2/13 (15%) OvFC] either by surgical pathology or fine needle aspiration cytopathology examination. All aspects of this study were approved by the Cleveland Clinic Institutional Review Board under protocol # 16-099 (project approval date 01/24/2016). Ethical practices were followed throughout.

### Tissue microarray

Thyroid tissue microarrays (TMAs) were constructed from original formalin-fixed, paraffin-embedded (FFPE) tissue blocks, as previously described [27]. In brief, regular 5-μm sections were made and stained with Hematoxylin and Eosin (H&E) to confirm previously rendered histologic diagnoses based upon current WHO classification [25]. Selected areas were marked on the H&E stained slides as a guide for TMA construction by one pathologist (CS). Focused areas from the central region of each submitted tumor were identified for inclusion in the array based upon histology being representative of the entire lesion, tissue being representative of well-fixed regions of the paraffin blocks, and tumor tissue viability (excluding areas of necrosis). Focused areas of benign thyroid tissue taken from between hyperplastic / adenomatoid nodules were selected as control cores in the cases of nodular hyperplasia. Tissue cylinders with a diameter of 1.2 mm were punched from each donor tissue block and brought into the recipient paraffin block using “Pathology Devices TMArrayer” (Pathology Devices Inc., Westminster, MD). Two cores from each case i.e. NH or thyroid follicular cell-derived carcinomas were arrayed with 1.8 mm spacing. A total of three TMAs were created; TMA1 included the NH and PTC cases, TMA2 housed the FC and OvFC cases, and TMA3 had the UDC cases. The first core of each TMA was made from normal liver tissue to allow proper orientation by the scoring pathologists. Slides were cut and stained with H&E to ensure that the all of the desired tissue cores were present.

### Immunohistochemistry

Immunohistochemistry (IHC) staining for cMYC was performed on the Ventana Benchmark Ultra automated immunostainer (Ventana Medical Systems (VMS), Tucson AZ). Formalin-fixed, paraffin-embedded TMA sections as well as selected whole tissue sections [from UDC cases that had concomitant well differentiated carcinomas (10) or PTC preexisting UDC (1)] were cut at 4 μm. Sections were treated with online deparaffinization, followed by online epitope retrieval using a high pH Tris-based solution (VMS Ultra CC1) for 64 min at 100 °C. The slides were incubated with the anti-cMYC primary antibody [rabbit monoclonal primary antibody (Y69), Abcam (Cambridge, MA)] prediluted to 1: 50 for 32 min with no heat. Localization of the antigen-antibody complex was achieved using the VMS OptiView DAB detection kit (biotin-free polymer system). The complex is visualized with hydrogen peroxide substrate and diaminobenzidine tetrahydrochloride (DAB) chromogen. Appropriate positive and negative controls were included.

For BRAF^V600E^ mutation, IHC staining was performed on formalin-fixed, paraffin-embedded tissues cut in 4 μm sections on the Ventana Benchmark Ultra automated immunostainer (VMS, Tucson AZ). Online deparaffinization was followed by online epitope retrieval using “VMS Ultra CC1” solution for 64 min at 100 °C. The slides were incubated with the anti-BRAF ^V600E^ primary antibody [mouse monoclonal (VE1), Spring Bioscience, Pleasanton CA] at 1:175 dilution for 16 min at 37 °C. Localization of the antigen-antibody complex was achieved using the VMS OptiView DAB detection kit and VMS OptiView Amplification kit. Appropriate positive and negative controls were included.

Evaluation of the pattern of immunoreactivity, percentage of cells staining, and staining intensities for cMYC and BRAF^V600E^ were performed by two independent pathologists (HS & CS). Scoring systems for proportion of positive cells and staining intensity were adapted from previously published investigations [21, 22, 24]. The percentage of cells staining was graded on a scale of 1–4 [1 = 10–24%; 2 = 25–49%; 3 = 50–74%; and 4 = ≥ 75%]. For cMYC, the expression was almost exclusively seen in a nuclear fashion in both thyroid carcinomas and nodular hyperplasias. The cut off for a negative result was ≤9% cell staining. Staining intensity was scored on a scale of +1 to +3 [+1 = weak, +2 = intermediate, +3 = strong]. Both percentage of cells staining and intensities of staining were used to define weakly versus strongly positive cMYC cases as well as to set a cutoff for BRAF positivity. Weakly positive cases cMYC cases were those with ≥1 (percentage scale) and +1 (intensity scale) reading, and strongly positive cases were those with ≥1 (percentage scale) and ≥ + 2 (intensity scale). For BRAF, the expression was almost exclusively seen in a cytoplasmic, granular fashion in thyroid carcinomas. Positive cases were defined as ≥3 (percentage scale) and ≥ + 1 (intensity scale). Discrepant pattern, percentage and intensity results were resolved by simultaneous dual-headed microscope review and consensus agreement of the two independent pathologists.

### Statistical analysis

Comparison of distributions of clinicopathologic variables among different histologies in the study was performed using two-tailed Student’s *t* test (Microsoft Excel, 2010, Seattle, WA) for continuous variables (e.g. age), and Fisher’s exact test (GraphPad Prism software, GraphPad, La Jolla, CA) for categorical variables (e.g. gender). Mean values of cMYC expression were compared using two-tailed Student’s *t* tests. Comparison of distributions of cMYC expression among all histologic types was performed by an exact Kruskal-Wallis test. Pairwise comparisons of distributions of cMYC expression among pairs of histologies, as well as clinicopathologic variables (e.g. age, tumor size) for each histologic type of thyroid carcinoma were performed by an exact Mann-Whitney test (unless otherwise specified). *P* values <0.05 were regarded as statistically significant.

## Results

The clinicopathologic characteristics of the patients examined are detailed in Table 2. UDC occurred in persons of more advanced age (mean of 69 years) than did NH / PTC / FC (*p* ≤ 0.0002). Of note, OvFC patients showed an older mean age than NH/ PTC / FC; however, this age difference was not statistically significant. All UDC patients were considered to have pT4 tumors based upon current AJCC classification. In general, the sample shows a female predominance of thyroid follicular cell-derived carcinomas (2-3: 1). There was no statistically significant difference in gender distribution of cases between NH and any of the carcinoma groups.

**Table 2:** Clinicopathologic characteristics of the study groups

Clinicopathologic characteristics	NH (*n* = 25)	PTC (*n* = 25)	FC (*n* = 25)	OvFC (*n* = 25)	UDC (*n* = 22)	*P*
Age Range (mean ± SD)	20 – 79 (51 ± 17.3)	20 – 72 (49 ± 15.1)	15 – 80 (49 ± 20.2)	27 – 87 (61 ± 16.2)	47 – 84 (69 ± 12.01)*	0.0002*
Gender	22 F (88%) 3 M (12%)	16 F (64%) 9 M (36%)	19 F (76%) 6 M (24%)	17 F (68%) 8 M (32%)	16 F (73%) 6 M (27%)	NS
Tumor stage	N/A	(*n* = 25)	(*n* = 25)	(*n* = 25)	(*n* = 22)	
pT1		9	4	4	0	N/A
pT2		3	14	8	0	N/A
pT3		13	7	11	0	N/A
pT4		0	0	2	22^#^	N/A

Undifferentiated carcinomas (UDC) occurred in an older age group than all other categories [*p* ≤ 0.0002(^*^)]. By AJCC definition, UDC cases are considered pT4 tumors (^#^). The mean of ages among different groups was compared using two-tailed *t* test. Differences between the gender distributions of cases were analyzed by Fischer’s exact test

***NH*** nodular hyperplasia, ***PTC*** papillary thyroid carcinoma, ***FC*** follicular carcinoma, ***OvFC*** oncocytic variant of follicular carcinoma, ***UDC*** undifferentiated carcinoma, ***F*** female, ***M*** male, ***N/A*** not applicable, ***NS*** non-significant

### Immunohistochemistry for cMYC protein

**Fig. 1:**
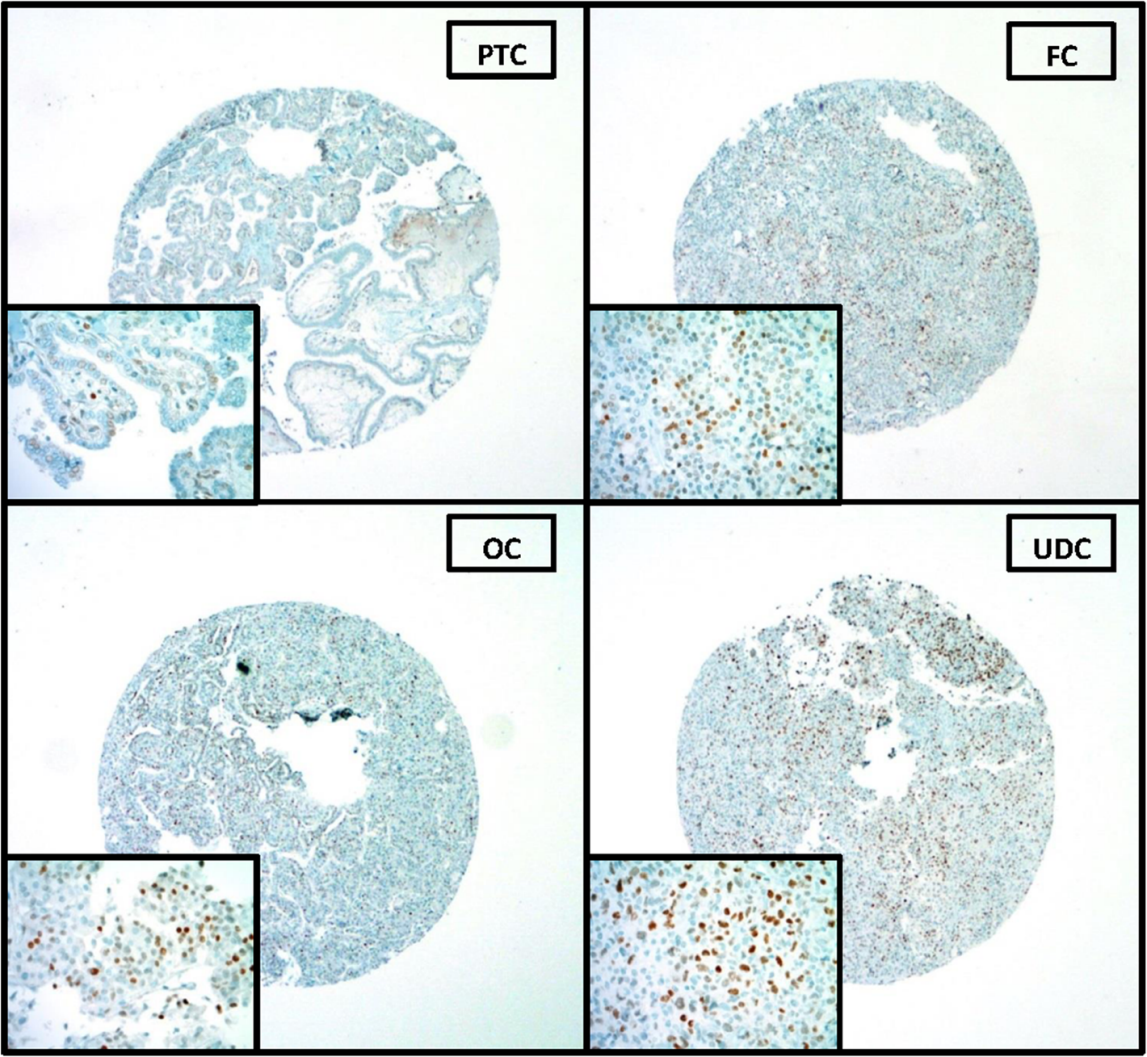
cMYC nuclear expression by immunohistochemistry in a spectrum of follicular cell-derived carcinomas (representative TMA images). cMYC expression was observed in a nuclear pattern. The original magnification of the whole TMA cores was 4X, with the photomicrographic insets taken at 40X. In this image, PTC showed >25% cells staining weakly for cMYC (scores 2 & +1, respectively), FC had a score of 2 & +2, OvFC had 3 & +2 score and UDC showed diffuse, mostly strong staining with a score of 4 & +3

cMYC expression was investigated in a spectrum of 94 follicular cell-derived thyroid carcinomas from 94 separate patients using an anti-MYC rabbit monoclonal antibody (Y69). In our study, there was a wide range of cMYC nuclear expression from absent to moderate among most study groups. UDC was the only group that showed strong nuclear cMYC expression (Fig. 1). In NH, weak cMYC expression (proportion of cells positive without regard to intensity) was noted with a mean of 0.64 ± 0.7. In sharp contrast, UDC showed the highest mean for cMYC expression (proportion of cells positive without regard to intensity) (1.7 ± 1.3) compared to all histologic types of thyroid carcinomas. The cMYC expression in UDC was significantly higher than PTC and FC (all *p* < 0.05) (Table 3). Thirteen out of the 22 UDC (nearly 60%) cases were associated with well differentiated thyroid carcinomas. Out of these 13 UDC cases, 8 cases showed cMYC staining on UDC TMA (62%), which represented 50% of the total number of cMYC positive cases in the UDC group. Per pathology reports, all of those 8 positive cases had either a concomitant PTC (7/8) or OvFC (1/8).

**Table 3:** cMYC nuclear expression in nodular hyperplasias and follicular cell-derived carcinomas

cMYC expression	NH (*n* = 25)	PTC (*n* = 25)	FC (*n* = 24)^a^	OvFC (*n* = 24)^b^	UDC (*n* = 21)^c^
Negative	12	19	18	12	5
Weak positive	11	5	5	7	4
Strong positive	2	1	1	5	12
Total positive	13	6	6	12	16
*P* value total positives (UDC vs other)	0.13	**0.0009**	**0.0009**	0.12	
*P* value strong positives (UDC vs other)	**0.0004**	**0.0001**	**0.0001**	**0.02**	

cMYC nuclear expression was compared between different groups, as shown above using Fisher’s exact test. ^a, b, c^ One case was excluded from each of the FC, OvFC, and UDC categories secondary to TMA tissue loss

***NH*** nodular hyperplasia, ***PTC*** papillary thyroid carcinoma, ***FC*** follicular carcinoma, ***OvFC*** oncocytic variant of follicular carcinoma, ***UDC*** undifferentiated carcinoma

Statistically significant *P* values are in bold

**Fig. 2:**
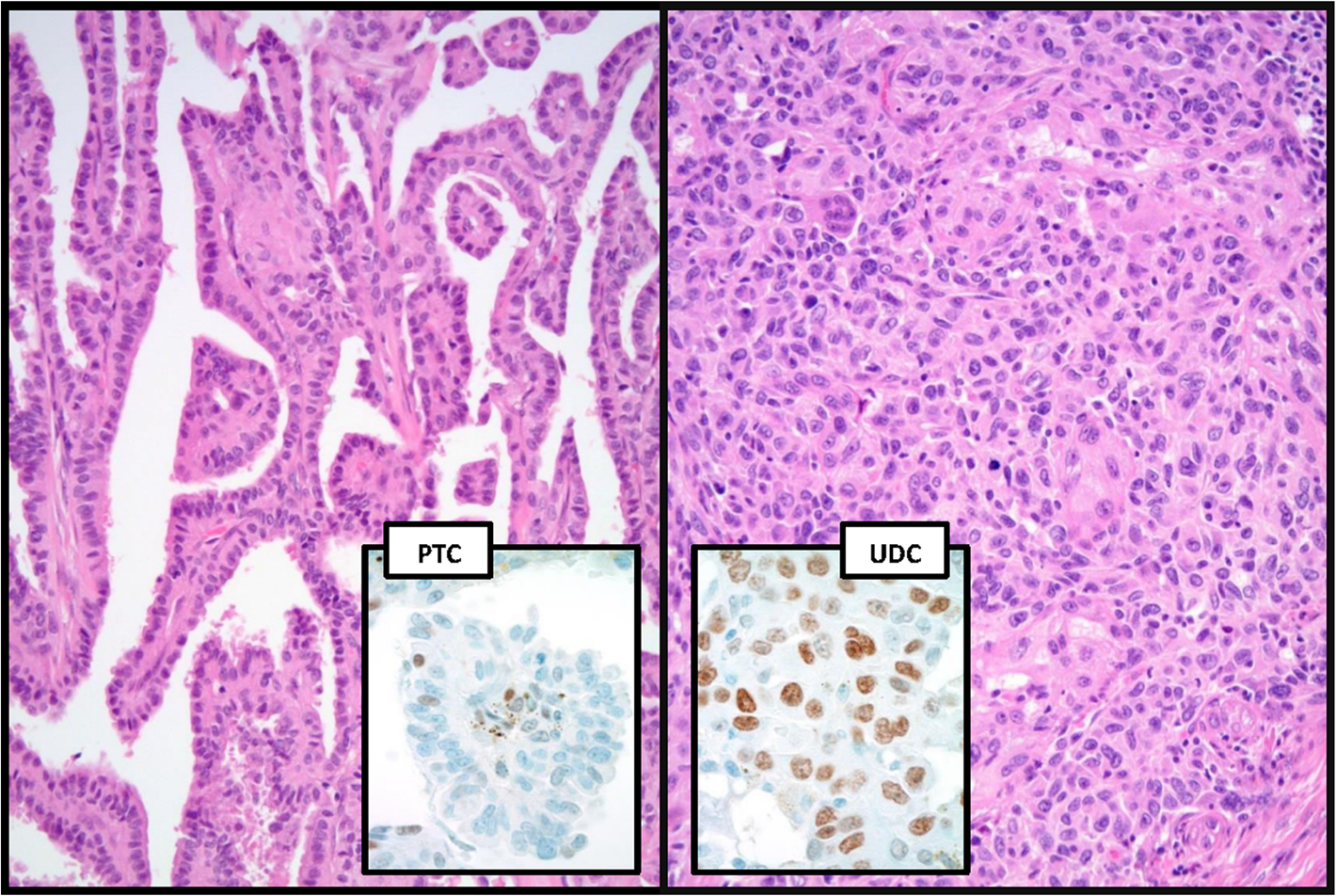
cMYC overexpression in a UDC developing out of a PTC (representative whole slide images). cMYC nuclear overexpression as seen in one of the UDC cases concomitantly developing out of a PTC. The PTC case showed >25% cells staining weakly for cMYC (scores 2 & +1, respectively) with UDC developing in vicinity showing 3 & +2 score. H&E background photomicrographs were originally taken at 20× magnification, and the cMYC IHC inserts were taken at 60X magnification

Whole tissue sections available for cMYC IHC staining of the well differentiated carcinomas occurring concomitantly with UDC cases (10/13) or as a preexisting diagnosis (PTC, 1/13) were even more informative than the TMA. Those 11 cases included 9 PTC cases and 2 OvFC cases. Almost all PTC cases (8/9) whether classic (5/9) or with tall cell features (3/9) showed cMYC expression, with loss of expression in follicular variant PTC (1/9). The two OvFC cases did not show cMYC expression. The UDC component in those sections with concomitant well differentiated carcinomas showed cMYC staining pattern and intensity similar to that appreciated in the TMA. In addition, expression was significantly higher than the accompanying well differentiated carcinomas (Fig. 2).

When the study groups were stratified based upon IHC staining intensity of cMYC, nearly half of the NH cases (44%) stained weakly for cMYC with only 2 cases (8%) showing moderate cMYC staining intensity. Most PTC and FC cases did not show any cMYC nuclear staining, with 20% in each category staining weakly for cMYC, and only 4% showing moderate cMYC staining. OvFC showed a distribution for cMYC staining intensities similar to NH but with more cases (21%) showing moderate cMYC intensity. Unlike the other thyroid carcinomas, UDC showed the greatest number of cases with strong cMYC intensity (+2 and +3). In addition, UDC was the only histologic type to show strong (+3) nuclear cMYC intensity. Comparison of the distributions of cMYC expression among tumor histologies showed a statistically significantly difference (*p* < 0.0001). Three pairwise comparisons between UDC and other tumor histologies were performed to determine the histologies in which significant differences in distributions of cMYC expression existed. UDC showed significantly higher cMYC expression than all other carcinoma types (PTC, FC, and OvFC) (all *p* < 0.005).

### cMYC Immunostaining and clinical data

cMYC expression was stratified in the carcinoma groups according to pathologic tumor staging “pT”, as defined by AJCC parameters. Given the small number of cases per group and the uneven distribution of pT stages in each group, no statistically significant correlations were demonstrated between cMYC expression and stage. For well differentiated types of thyroid carcinomas (PTC, FC, and OvFC), there was a non-significant trend toward lower cMYC expression from stages pT1 through pT3. Of note, a trend toward somewhat different cMYC staining was noted in the higher stage OvFC cases (pT3 and pT4). In the pT3 cases there was 40% (4/10) cMYC positivity, all of which was weak positivity. In contrast, the two pT4- OvFC cases showed strong cMYC positivity. Of note, patients with UDC > 20 mm had statistically significant higher cMYC intensities than those with smaller tumors (< 20 mm). In addition, 62% of UDC cases (8/13) that had a well-differentiated precursor lesion stained positive for cMYC. No significant differences in cMYC expression were found in any of the tumor types when comparisons with patient ages, or tumor sizes (other than UDC) were performed [Exact Mann Whitney].

### Immunohistochemistry for BRAF^V600E^ mutation

BRAF^V600E^ mutation was assessed in 96 follicular cell-derived thyroid carcinomas from 96 separate patients using an anti- BRAF^V600E^ mouse monoclonal antibody (VE1). Only PTC and UDC showed significant numbers of BRAF^V600E^ mutation positive cases, 15/25 (60%) for PTC and 11/21 (52%) for UDC, respectively (Table 4). Historical PCR results [224 base pair product including codon 600 with exon 15, Applied Biosystems; Foster City, CA] had previously confirmed 6 of 14 PTC cases positive for mutation. All PCR BRAF mutant cases were confirmed positive by TMA IHC. Only one case of OvFC showed the BRAF^V600E^ mutation. No BRAF^V600E^ mutation was detected in NH or FC cases. The relationship between cMYC expression and BRAF^V600E^ mutation status in PTC and UDC cases were independently examined. All the PTC cases that stained for cMYC by IHC harbored the BRAF^V600E^ mutation (*n* = 6), which represented 40% of all BRAF^V600E^ mutation positive PTC cases (6/15). No cMYC IHC staining was documented in BRAF^wt^ cases. For UDC cases, cMYC expression was distributed almost equally between UDC cases regardless of BRAF mutation status; 8/11 in BRAF^V600E^ mutation positive cases vs. 8/10 in BRAF^wt^ cases.

**Table 4:** BRAF^V600E^ expression in thyroid nodular hyperplasias and follicular cell-derived carcinomas

BRAF^V600E^ expression	NH (*n* = 25)	PTC (*n* = 25)	FC (*n* = 25)	OvFC (*n* = 25)	UDC (*n* = 21)^d^
Positive	0	15	0	1	11
Negative	25	10	25	24	10
*P* value^a^ (NH vs. other)		**0.0001**	1	1	**0.0004**
*P* value^b^ (PTC vs. other)			**0.0001**	**0.0001**	0.77
*P* value^c^ (UDC vs. other)			**0.0001**	**0.0004**	

BRAF^V600E^ expression was compared between different groups, as shown above (^a^ NH versus thyroid carcinomas, ^b^ PTC versus other thyroid carcinomas, ^c^ UDC versus FC and OC), using Fisher’s exact test. ^d^ One case was excluded from UDC category secondary to TMA tissue loss

***NH*** nodular hyperplasia, ***PTC*** papillary thyroid carcinoma, ***FC*** follicular carcinoma, ***OvFC*** oncocytic variant of follicular carcinoma, ***UDC*** undifferentiated carcinoma

Statistically significant *P* values are in bold

## Discussion

Herein, we conducted an extensive literature review for relevant IHC characterization studies performed since 1990 for cMYC protein expression in thyroid follicular cell-derived carcinomas. Some disparities regarding cMYC expression (staining patterns, sensitivities, and/or intensities) in thyroid follicular cell-derived carcinomas were noted in these references (see Table 1 for details). Studies have shown nuclear (mostly) and cytoplasmic (occasionally) expression patterns for cMYC. Of note, older studies failed to show nuclear IHC immunoreactivity for cMYC [14, 16–18]. It is well known that cMYC exerts its oncogenic potential through transcriptional deregulation, mostly activation, of many downstream genes which are involved in cellular proliferation, differentiation, and apoptosis [28–30]. Though several cMYC variants have been previously identified, most of them retain the nuclear localization signal (NLS) [31, 32]. Interestingly, there have been reports of cytoplasmic localization of cMYC, mostly in differentiated cells [33, 34]. Studies published for cytoplasmic cMYC expression in tumors have shown discordance pertaining to cMYC localization among histologic patterns of non-neoplastic and neoplastic tissues [35, 36]. Similar to our findings, these studies relied upon immunohistochemical evaluation without cMYC protein characterization. Some authors have suggested that a truncated cMYC isoform that is localized to the cytoplasm has no detectable effect on cell proliferation or survival [32].

Studies investigating cMYC expression in UDC are very limited. The only study with sizeable numbers of patients (number of cases, *n* = 22) showed 59% cMYC immunoreactivity, less than the 76% shown in our study. This manuscript did not comment on staining intensity [22].

Using a specific cMYC monoclonal antibody (Y69, rabbit monoclonal, Abcam), we confirmed, almost exclusively, a nuclear staining pattern for cMYC in thyroid follicular cell derived tumors. Contrary to previous studies (Table 1), results of cMYC expression in well differentiated carcinomas appear different with fewer positive cases in our work. Interestingly, cMYC expression was found to be significantly higher in UDC compared to nodular hyperplasias and well-differentiated carcinomas (PTC/FC/ OvFC) [all *p* < 0.05]. The sensitivity of the anti-cMYC monoclonal antibody used in the current study appears higher than that of the antibodies used previously. In the current study, the clinicopathologic data showed that 13 out of 22 UDC cases had precursor well-differentiated thyroid carcinomas, 62% of them (8/13) exhibited nuclear *cMYC* expression. Interestingly, 50% of the cMYC positive cases in UDC group (8/16) did not have any associated precursor lesion per pathology reports. Our data showed that undifferentiated carcinomas larger than 20 mm showed higher cMYC intensities (≥1+). A finding such as this has not been previously reported.

In the current study we used TMA for cMYC IHC staining. One concern that may arise is that UDCs may show heterogeneous protein expression levels in various areas of a single neoplasm. To overcome that problem and to elucidate a role of cMYC in thyroid tumorigenesis, we stained whole tissue sections from a subset of the 13 UDC cases that were documented to have either concomitant (10/13) or precursor (1/13) well-differentiated thyroid carcinomas. The results of cMYC IHC staining were informative and emphasize the importance of adequate tissue sampling in molecular studies. Those 11 cases included 9 PTC cases (with only 7 UDC component represented on the same tissue section) and 2 OvFC cases. In a sharp contrast to the PTC group (25 cases) in the TMA, almost all PTC cases (8/9) whether classic (5/9) or with tall cell features (3/9) that developed into UDC showed stronger and more diffuse cMYC expression. Only one case of PTC, follicular variant, did not show cMYC expression (1/9). Interestingly, the two OvFC cases preexisting UDC did not show cMYC expression. The UDC component associated with preexisting PTC (7 cases) showed cMYC overexpression in 100% of the cases and in one case (1/2) associated with preexisting OvFC. In addition, the pattern and intensity of cMYC expression for UDC in whole tissue sections was similar to that interpreted from the TMA cores.

BRAF^V600E^ mutation has been reported to occur almost exclusively in PTC and PTC-derived UDC, while BRAF^V600E^ mutations are known to be uncommon in FC and other types of thyroid carcinomas [3, 37]. We investigated the correlation between cMYC expression and BRAF status in PTC and UDC cases using a specific BRAF^V600E^ mutation monoclonal antibody. Our findings are similar to what have been previously published in the literature regarding BRAF^V600E^ mutation in thyroid follicular cell-derived carcinomas. We found BRAF^V600E^ to be expressed almost exclusively in PTC and UDC cases. In our study, more than half of the BRAF^V600E^ positive UDCs with preceding or concomitant well differentiated carcinomas showed diffuse, nuclear cMYC overexpression. We did not demonstrate a linear correlation between BRAF^V600E^ mutation and cMYC expression in UDC cases, either in those cases that seemed to arise in association with well differentiated carcinoma or in those that appeared de novo. Interestingly, all cases of PTC that were weakly positive for cMYC (6/25) harbored the BRAF^V600E^ mutation. A recent manuscript based upon multiplatform ‘omics’ molecular analysis of nearly 500 PTCs from The Cancer Genome Atlas (TCGA) Research Network categorized PTCs into two broad categories, a well differentiated RAS-like (RL) type with predominance of follicular variant histology, and a more heterogeneous, less differentiated BRAF^V600E^-like (BVL) type with classic and tall cell features [38]. In light of the TCGA study results, it could be inferred that some UDCs with preceding or concomitant PTCs could possess antecedent BRAF^V600E^ mutations either prior to or simultaneously with cMYC overexpression, the latter of which appears uniformly present in UDCs on our data. Further investigations of relationships between BRAF^V600E^ and cMYC in UDCs using refined molecular techniques and larger cohorts (samples from multiple institutions) are recommended.

Prior studies have analyzed PDC and UDC via next generation sequencing (NGS) to characterize genomic and/or transcriptomic landscape for these aggressive entities. Multiple candidate genes involved in signal transduction, cell cycle regulation, and DNA repair had been identified e.g. *EGFR*, *ATM*, and *TP53* [39, 40]. Noticeably, wide genomic analysis of variable numbers of UDC cases have failed to show mutations in cMYC. Prior reports based upon both thyroid cancer cell lines and animal model studies have suggested that cMYC plays a role in thyroid carcinogenesis and progression from well- to less-differentiated carcinomas [41, 42]. It is postulated that cMYC gene deregulations themselves (e.g. translocations or amplifications) are not the sole mechanisms for cMYC overexpression and subsequent thyroid carcinoma progression [13, 43]. It seems likely that other pathways / factors are associated with the increased mRNA levels and levels of MYC protein expression which have been documented to occur with increasing histologic aggressiveness and dedifferentiation in thyroid carcinomas [43, 44]. In our study, there was a concomitant expression/overexpression of cMYC in well differentiated thyroid carcinomas that developed into UDC. The overexpression of cMYC in UDC cases suggests a role for cMYC in the multi-stage process of carcinogenesis. Based on our findings, extensive tissue sampling with adequate representation for cMYC IHC staining in cases of PTC is recommended. In association with other mutations known to occur in UDC e.g. *EGFR*, *ATM*, or *TP53,* cMYC may be a potential biomarker useful for diagnostic work up of well differentiated thyroid carcinomas with aggressive features. Inclusion of poorly differentiated carcinomas in additional studies might provide additional insight at an intermediate stage along the spectrum of disease differentiation.

## Conclusions

In summary, we demonstrated a nuclear staining pattern of cMYC IHC in TMAs of follicular cell-derived thyroid carcinomas. cMYC expression in undifferentiated thyroid carcinomas was statistically significantly greater than in NH and well-differentiated carcinomas. cMYC positivity was identified in UDC cases (16/21, 76%) with strong positivity in more than half (57%) of all UDC cases. In addition, UDC cases that developed in association with or out of well-differentiated thyroid carcinomas showed frank overexpression of cMYC upon dedifferentiation. These findings might suggest a possible role for cMYC in thyroid carcinogenesis and dedifferentiation, a concept that is supported by both TMA and whole tissue section IHC in the current investigation. Additional specific next generation sequencing testing and / or mRNA studies of cMYC could prove valuable; however, such testing is beyond the scope of our investigations. UDC is an aggressive disease with limited therapeutic options and uniformly dismal associated clinical outcomes. Based upon our results, clinical trials of targeted anti-cMYC therapies might be considered in patients with UDC whose tumors can be demonstrated to express cMYC by IHC. Clinical trials with targeted anti-MYC therapies might provide additional avenues for therapy to UDC patients with limited therapeutic options.

## Abbreviations

FC: Follicular carcinoma
FFPE: Formalin-fixed, paraffin-embedded
IHC: Immunohistochemistry
NH: Nodular hyperplasia
OvFC: Oncocytic variant of FC
PTC: Papillary thyroid carcinoma
TMA: Tissue microarray
UDC: Undifferentiated carcinoma
